# Novel Anthranilamide-Based FXa Inhibitors: Drug Design, Synthesis and Biological Evaluation

**DOI:** 10.3390/molecules21040491

**Published:** 2016-04-14

**Authors:** Wenzhi Wang, Jing Yuan, Xiaoli Fu, Fancui Meng, Shijun Zhang, Weiren Xu, Yongnan Xu, Changjiang Huang

**Affiliations:** 1 School of Pharmaceutical Engineering, Shenyang Pharmaceutical University, Shenyang 110016, China; wangwenzhi870507@163.com; 2 Tianjin Key Laboratory of Molecular Design and Drug Discovery, Tianjin Institute of Pharmaceutical Research, Tianjin 300193, China; yuanj@tjipr.com (J.Y.); fuxl@tjipr.com (X.F.); mengfc@tjipr.com (F.M.); zhangsj@tjipr.com (S.Z.); xuwr@tjipr.com (W.X.)

**Keywords:** thrombosis, FXa inhibitor, anthranilamide-based FXa inhibitors, docking simulation

## Abstract

Factor Xa (FXa) plays a significant role in the blood coagulation cascade and it has become a promising target for anticoagulation drugs. Three oral direct FXa inhibitors have been approved by the FDA for treating thrombotic diseases. By structure-activity relationship (SAR) analysis upon these FXa inhibitors, a series of novel anthranilamide-based FXa inhibitors were designed and synthesized. According to our study, compounds **1a**, **1g** and **1s** displayed evident FXa inhibitory activity and excellent selectivity over thrombin in *in vitro* inhibition activities studies. Compounds **1g** and **1s** also exhibited pronounced anticoagulant activities in *in vitro* anticoagulant activity studies.

## 1. Introduction

Thromboembolic diseases such as myocardial infarction (MI), pulmonary embolism (PE), deep vein thrombosis (DVT) and ischemic strokes are the major causes of mortality all over the world in the 21st century [1,2,3]. The typical method for treating and preventing these diseases is to use anticoagulant drugs [4]. For example, vitamin K antagonists (VKAs), low-molecular-weight heparins (LMWHs) and warfarin are validated in the prevention and treatment of these thrombotic disorders [5], but they still have many shortcomings in clinical applications including the inconvenience of frequent monitoring, interactions with many drugs and food, and slow onset action [6,7]. These shortcomings further accelerate the need for development of new oral anticoagulants with efficacy and safety.

**Figure 1:**
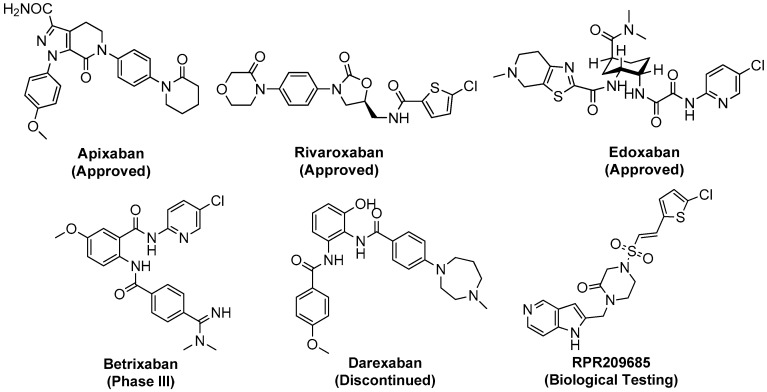
Oral direct FXa inhibitors.

Factor Xa (FXa), which is located at the junction of the intrinsic and extrinsic pathways in the coagulation cascade and catalyzes the conversion of prothrombin to thrombin, plays a pivotal role in the blood coagulation cascade and has become a promising target for anticoagulation effects [4,8]. It is well demonstrated that selective FXa inhibitors can decrease the generation of thrombin without affecting the existing thrombin level which means that selective FXa inhibitors can play an ideal antithrombotic effects without influencing normal hemostasis and decrease the risk of bleeding [9,10,11]. Three oral direct FXa inhibitors have been approved by the FDA for treating thrombotic diseases and several FXa inhibitors have entered the stage of clinical research or biological testing (Figure 1) [2,12,13,14]. However, they still have many drawbacks, such as drug interactions, narrow indications and long treatment duration [15]. Therefore, new antithrombotic drugs still need to be developed for addressing these issues.

**Figure 2:**
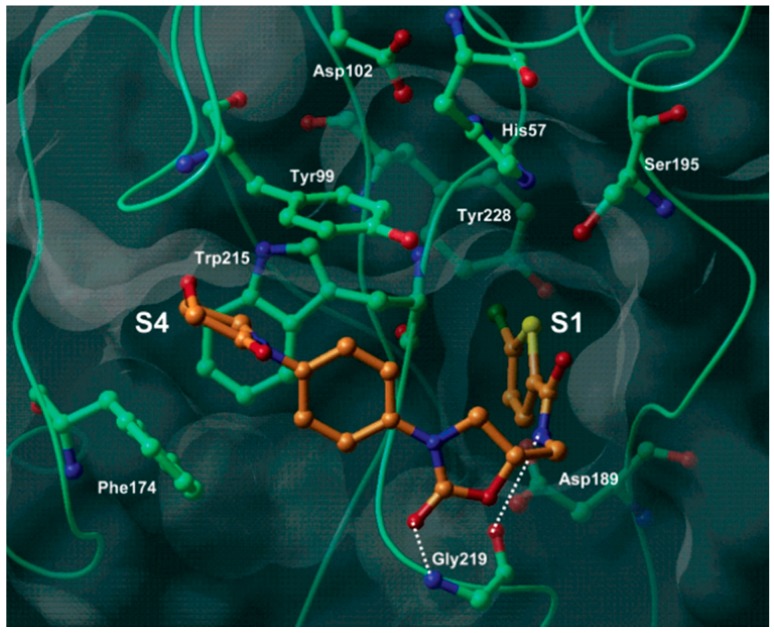
X-ray crystal structure of rivaroxaban in complex with human FXa.

**Figure 3:**
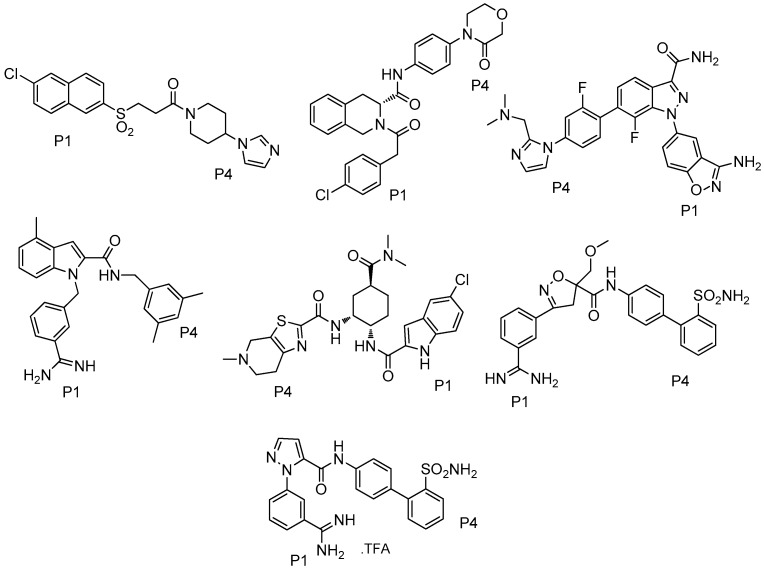
Examples of the molecules with typical structure.

From the structures above, FXa inhibitors have three components which make up the pharmacophore: core scaffold, P1 and P4. This typical structure can help these molecules combine with FXa, P1 locates at the S1 pocket and P4 locates at the S4 pocket (Figure 2) [16]. Presented if Figure 3 here are several examples can help us understand this more clearly [17,18,19,20,21,22,23].

**Figure 4:**
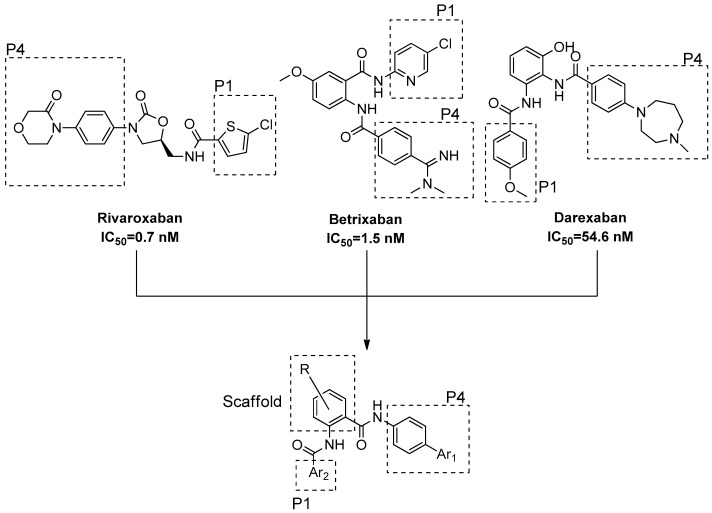
Design of anthranilamide-based FXa inhibitors.

Interestingly, we found that for both rivaroxaban and betrixaban employed a carboxamide group to connect the scaffold, P1 and P4. Based on analysis of the X-ray crystal structure of rivaroxaban complexed with human FXa, it was found that there are two hydrogen bonds formed between rivaroxaban and the residue Gly219 in FXa [12]. Similarly, betrixaban possesses two hydrogen bonds with residues Gly218 and Gly216 in FXa [24], so we hypothesized that the key factor for FXa inhibitors docked to human FXa might be the carboxamide group. Based on the structures of rivaroxaban, betrixaban and darexaban, we have designed a series of novel FXa inhibitors employing anthranilamide as the scaffold (Figure 4). Through metabolism of rivaroxaban *in vivo* [25], we can find that the main metabolic pathway is the hydrolysis of the amide bond. We reversed the carbonyl and amino connection order of betrixaban with the expectation that the designed compounds may display different toxicity data or participate in different metabolic pathways in humans.

## 2. Results and Discussion

### 2.1. Synthesis

**Scheme 1:**
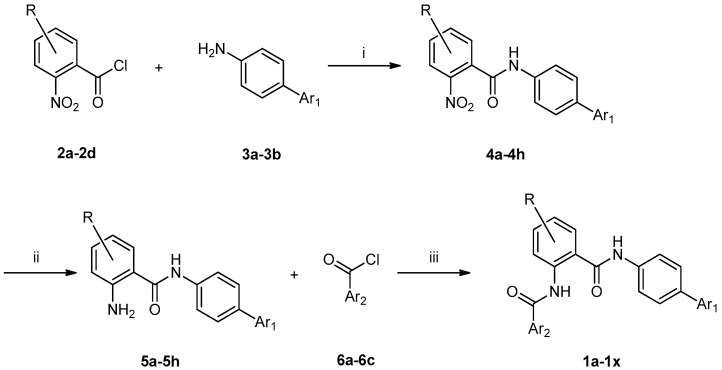
Synthetic route of compounds **1a**–**1x**. *Reagents and conditions*: (i) THF, K_2_CO_3_, DMAP, reflux, 2 h; (ii) Zn, NH_4_Cl, H_2_O, THF, MeOH, 40 °C, 2 h; (iii) THF, K_2_CO_3_, DMAP, reflux, 4 h.

The synthetic routes used in this study are illustrated in Scheme 1. All the starting material are commercial available. As shown in Scheme 1, the acylation [26] of **3a**–**3b** respectively with a series of *o*-nitrobenzoyl chloride **2a**–**2d** provided **4a**–**4h**, which were subsequently reduced with Zn, NH_4_Cl to provide the corresponding anilines **5a**–**5h** in satisfied yields. Acylation [26] of **5a**–**5h** respectively with **6a**–**6c** yielded the target compounds **1a**–**1x**.

### 2.2. Biological Activities and Discussion

#### 2.2.1. *In Vitro* Inhibition Activity Studies on FXa

**Figure 5:**
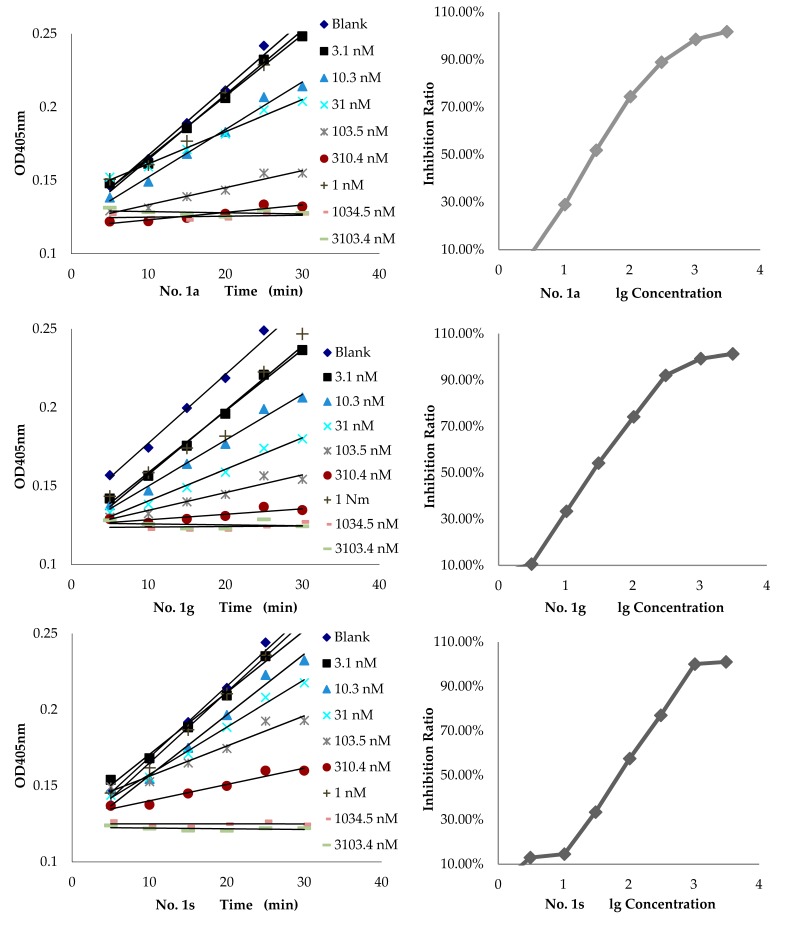
The inhibition profile figures of **1a**, **1g** and **1s**.

All the targeted compounds were evaluated *in vitro* for investigating their FXa inhibitory activity, using rivaroxaban as the positive control in this assay. The assay results (Table 1) showed that several designed compounds exhibited inhibitory activity against FXa with IC_50_ values at the nanomole level from 951.3 to 23.0 nM. In particular compound **1g** was the most promising FXa inhibitor in this series with an IC_50_ value of 23.0 nM. The results indicated that the compounds with a 3-methyl-substituted scaffold **(1j**–**1l**, **1v**–**1x**) possessed relative poor inhibitory activity against FXa, with IC_50_ values at a micromole level no matter what kind of Ar_1_ and Ar_2_ in Table 1 they linked with. When the Ar_1_ group was pyridin-2(1*H*)-one, the compounds (**1a**–**1l**) demonstrated that the affinity with FXa ranking was electron withdrawing group substituted scaffold < electron donating group scaffold < non-substituted scaffold. Through Table 1 can also be found that using 5-chlorothiophene as P1 (Ar_2_) the anticoagulant activity was much better than in other cases (**1a** = 30.9 nM, Ki = 22.0 nM; **1g** = 23.0 nM, Ki = 16.4 nM; **1s** = 76.2 nM, Ki = 54.4 nM; Km = 2.4). We also show an inhibition profile figure of the most potent compounds **1a**, **1g** and **1s** in Figure 5.

#### 2.2.2. Selectivity *vs.* Thrombin and Prothrombin Time (PT) Assay

To evaluate the inhibitory activity against FXa of compounds **1a**, **1g** and **1s** more accurately, these compounds were chosen to assess degree of selectivity *versus* thrombin and the extension of the prothrombin time (PT). Compounds **1a**, **1g** and **1s** showed no inhibition effect on thrombin, with IC_50_ values far higher than 10 μM. They showed similar selectivity against thrombin as rivaroxaban which is far more than 6.9 μM [27]. The prothrombin time (PT) assay results are shown in Table 2, where compounds **1g** and **1s** also show good anticoagulant activity, judging by their 2 × PT value of 19.7 and 24.2 μM in rat plasma and 12.8 μM and 10.4 μM in human plasma.

#### 2.2.3. Docking Simulation Studies on the Interaction of Compounds **1g** and **1s** with FXa

**Figure 6:**
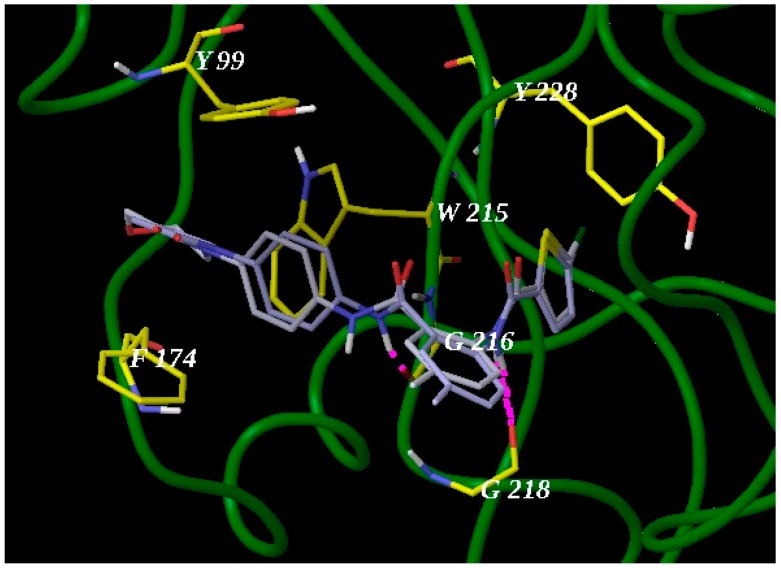
The interactions of compounds **1g** and **1s** with the active site of FXa.

Computer-based docking simulation studies were used to analyze the binding mode of compounds **1g** and **1s** at the active site of FXa. As shown in Figure 6, the pyridine moiety of **1g** and morpholino unit of **1s** are located at the sides of the phenyl groups of Tyr99 and Phe174 of the S4 aryl binding pocket, while its aryl ring is oriented perpendicularly, extending across the face of Trp215. Two hydrogen bonds are formed between **1g** and the residue Gly216 and Gly218 in FXa and one hydrogen bond is formed between **1s** and the amino acid Gly218 of FXa. In the S1 pocket of FXa, there is an interaction between the chlorine substituent of the thiophene moiety and the aromatic ring of Tyr228 at the bottom of the S1 pocket. They thus show similar interactions with FXa as rivaroxaban reported before [12].

## 3. Materials and Methods

### 3.1. General Information

Reagents and solvents were obtained from commercial suppliers and used as received without further purification. All reactions were monitored by thin layer chromatography. ^1^H-NMR spectra (400 MHz) were recorded for DMSO-*d*_6_ solutions on an AV400 NMR (Bruker, Billerica, MA, USA), MS were measured on a Finnigan LCQ Mass (Thermo Fisher Scientific, Cambridge, MA, USA), HRMS were measured on a miorOTOF-QII instrument (Bruker Daltonics, Billerica, MA, USA) and melting points (uncorrected) were determined on a YRT-3 Melting Point Tester (Precision Instrument of Tianjin University, Tianjin, China).

### 3.2. Chemistry

*2-Nitro-N-(4-(2-oxopyridin-1(2H)-yl)phenyl)benzamide* (**4a**). To a stirred solution of 1-(4-aminophenyl)pyridin-2(1*H*)-one (**3a**) (0.90 g, 4.8 mmol), K_2_CO_3_ (0.80 g, 5.8 mmol) and DMAP (0.05 g, 0.4 mmol) in THF (20 mL), solution of 2-nitrobenzoyl chloride (**2a**) (1.15 g, 6.24 mmol) in THF (5 mL) was added at room temperature and the mixture was refluxed for 2 h. The reaction mixture was cooled down to room temperature and concentrated under reduced pressure. Then water (100 mL) was added to the mixture and stirred for 10 min at room temperature. The resulting precipitate was collected by filtration. The reaction was monitored by TLC with EA. White solid product (1.46 g, 90%). MS: [M + H]^+^ 336.22. ^1^H-NMR: δ ppm 6.30 (t, *J* = 6.4 Hz, 1H), 6.47 (d, *J* = 9.2 Hz, 1H), 7.39 (d, *J* = 8.8 Hz, 2H), 7.49 (t, *J* = 4.4 Hz, 1H), 7.63 (d, *J* = 6.8 Hz, 1H), 7.76–7.80 (m, 4H), 7.89 (t, *J* = 7.6 Hz, 1H), 8.17 (d, *J* = 8.0 Hz, 1H), 10.84 (s, NH).

*5-Chloro-2-nitro-N-(4-(2-oxopyridin-1(2H)-yl)phenyl)benzamide* (**4b**). Compound **4b** was prepared from **3a** and 5-chloro-2-nitrobenzoyl chloride (**2b**) according to the procedure described for the preparation of **4a**. White solid product (1.64 g, 92%). MS: [M + H]^+^ 370.05. ^1^H-NMR: δ ppm 6.31 (t, *J* = 6.8 Hz, 1H), 6.47 (d, *J* = 9.2 Hz, 1H), 7.40 (d, *J* = 8.4 Hz, 2H), 7.50 (t, *J* = 2.4 Hz, 1H), 7.63 (d, *J* = 8.8 Hz, 1H), 7.76 (d, *J* = 6.8 Hz, 2H), 7.86 (d, *J* = 6.4 Hz, 1H), 8.00 (s, 1H), 8.20 (d, *J* = 8.8 Hz, 1H), 10.88 (s, NH).

*5-Methyl-2-nitro-N-(4-(2-oxopyridin-1(2H)-yl)phenyl)benzamide* (**4c**). Compound **4c** was prepared from **3a** and 5-methyl-2-nitrobenzoyl chloride (**2c**) according to the procedure described for the preparation of **4a**. White solid product (1.50 g, 89%). MS: [M + H]^+^ 350.42. ^1^H-NMR: δ ppm 2.43 (s, 3H), 6.31 (t, *J* = 6.4 Hz, 1H), 6.47 (d, *J* = 9.2 Hz, 1H), 7.38 (d, *J* = 8.8 Hz, 2H), 7.50 (t, *J* = 4.8 Hz, 1H), 7.55–7.64 (m, 3H), 7.76 (d, *J* = 9.2 Hz, 2H), 8.08 (d, *J* = 8.4 Hz, 1H), 10.78 (s, NH).

*3-Methyl-2-nitro-N-(4-(2-oxopyridin-1(2H)-yl)phenyl)benzamide* (**4d**). Compound **4d** was prepared from **3a** and 3-methyl-2-nitrobenzoyl chloride (**2d**) according to the procedure described for the preparation of **4a**. White solid product (1.54 g, 91%). MS: [M + H]^+^ 350.10. ^1^H-NMR: δ ppm 2.36 (s, CH_3_), 6.30 (t, *J* = 6.4 Hz, 1H), 6.46 (d, *J* = 9.2 Hz, 1H), 7.38–7.40 (m, 2H), 7.49 (t, *J* = 8.8 Hz, 1H), 7.62 (d, *J* = 8.8 Hz, 1H), 7.64–7.67 (m, 2H), 7.69–7.73 (m, 1H),7.77 (d, *J* = 8.8 Hz, 2H), 10.87 (s, NH).

*2-Nitro-N-(4-(3-oxomorpholino)phenyl)benzamide* (**4e**). Compound **4e** was prepared from 4-(4-aminophenyl)morpholin-3-one (**3b**) and **2a** according to the procedure described for the preparation of **4a**. White solid product (1.54 g, 93 %). MS: [M + H] ^+^ 342.09. ^1^H-NMR: δ ppm 3.71 (t, *J* = 4.8 Hz, CH_2_), 3.97 (t, *J* = 4.8 Hz, CH_2_), 4.19 (s, 2H), 7.37 (d, *J* = 8.8 Hz, 2H), 7.67 (d, *J* = 8.8 Hz, 2H), 7.74–7.78 (m, 2H), 7.87 (t, *J* = 7.6 Hz, 1H), 8.15 (d, *J* = 8.0 Hz, 1H), 10.72 (s, NH).

*5-Chloro-2-nitro-N-(4-(3-oxomorpholino)phenyl)benzamide* (**4f**). Compound **4f** was prepared from **3b** and **2b** according to the procedure described for the preparation of **4a**. White solid product (1.67 g, 94%). MS: [M + H]^+^ 376.05. ^1^H-NMR: δ ppm 3.72 (t, *J* = 10.0 Hz, 2H), 3.97 (t, *J* = 10.0 Hz, 2H), 4.19 (s, 2H), 7.38 (d, *J* = 8.4 Hz, 2H), 7.66 (d, *J* = 8.8 Hz, 2H), 7.84 (d, *J* = 8.8 Hz, 2H), 7.94 (s, 1H), 8.18 (d, *J* = 8.8 Hz, 2H), 10.77 (s, NH).

*5-Methyl-2-nitro-N-(4-(3-oxomorpholino)phenyl)benzamide* (**4g**). Compound **4g** was prepared from **3b** and **2c** according to the procedure described for the preparation of **4a**. White solid product (1.55 g, 90%). MS: [M + H]^+^ 355.99. ^1^H-NMR: δ ppm 2.47 (s, CH_3_), 3.71 (t, *J* = 4.8 Hz, CH_2_), 3.97 (t, *J* = 4.8 Hz, CH_2_), 4.19 (s, CH_2_), 7.37 (d, *J* = 8.8 Hz, 2H), 7.55 (d, *J* = 8.8 Hz, 2H), 7.57 (s, 1H), 7.67 (d, *J* = 8.8 Hz, 2H), 8.07 (d, *J* = 8.4 Hz, 1H), 10.66 (s, NH).

*3-Methyl-2-nitro-N-(4-(3-oxomorpholino)phenyl)benzamide* (**4h**). Compound **4h** was prepared from **3b** and **2d** according to the procedure described for the preparation of **4a**. White solid product (1.53 g, 89%). MS: [M + H]^+^ 356.19. ^1^H-NMR: δ ppm 2.36 (s, CH_3_), 3.72 (t, *J* = 4.8 Hz, CH_2_), 3.98 (t, *J* = 4.8 Hz, CH_2_), 4.20 (s, CH_2_), 7.38 (d, *J* = 8.8 Hz, 2H), 7.65–7.24 (m, 5H), 10.76 (s, NH).

*2-Amino-N-(4-(2-oxopyridin-1(2H)-yl)phenyl)benzamide* (**5a**). To a 250 mL round bottom flask, **4a** (1.40 g, 3.9 mmol), zinc powder (2.05 g, 31.2 mmol), NH_4_Cl (2.11 g, 39 mmol), methanol (30 mL), THF (30 mL) and water (15 mL) were added. The mixture stirred at 40 °C for 2 h. The reaction mixture was filtered, washed with DMF and the filtrate was concentrated under reduced pressure. Then water (200 mL) was added to the mixture and stirred for 0.5 h. The residue was filtered and washed with water to yield the title compound as a white solid product (1.13 g, 89%). The reaction was monitored by TLC with EA. MS: [M + H]^+^ 306.10. ^1^H-NMR: δ ppm 6.30 (t, *J* = 6.4 Hz, 1H), 6.34 (s, NH_2_), 6.46 (d, *J* = 8.8 Hz, 1H), 6.60 (t, *J* = 7.2 Hz, 1H), 6.76 (d, *J* = 8.4 Hz, 1H), 7.21 (t, *J* = 7.2 Hz, 1H), 7.33–7.36 (m, 2H), 7.49 (t, *J* = 8.8 Hz, 1H), 7.61–7.65 (m, 2H), 7.83 (d, *J* = 8.8 Hz, 2H), 10.14 (s, NH).

*2-Amino-5-chloro-N-(4-(2-oxopyridin-1(2H)-yl)phenyl)benzamide* (**5b**). Compound **5b** was prepared from **4b** according to the procedure described for the preparation of **5a**. White solid product (1.29 g, 91%). MS: [M + H]^+^ 340.10. ^1^H-NMR: δ ppm 6.30 (t, *J* = 5.2 Hz, 1H), 6.47 (d, *J* = 10.4 Hz, 1H), 6.48 (s, NH_2_), 6.78 (d, *J* = 9.2 Hz, 1H), 7.24 (d, *J* = 6.8 Hz, 1H), 7.35 (d, *J* = 8.8 Hz, 2H), 7.49 (t, *J* = 7.2 Hz, 1H), 7.63 (d, *J* = 8.4 Hz, 1H), 7.70 (s, 1H), 7.80 (d, *J* = 8.8 Hz, 2H), 10.24 (s, NH).

*2-Amino-5-methyl-N-(4-(2-oxopyridin-1(2H)-yl)phenyl)benzamide* (**5c**). Compound **5c** was prepared from **4c** according to the procedure described for the preparation of **5a**. White solid product (1.23 g, 92%). MS: [M + H]^+^ 320.04. ^1^H-NMR: δ ppm 2.22 (s, CH_3_), 6.11 (s, NH_2_), 6.30 (t, *J* = 6.4 Hz, 1H), 6.46 (d, *J* = 8.8 Hz, 1H), 6.68 (d, *J* = 8.4 Hz, 1H), 7.04 (d, *J* = 8.0 Hz, 1H), 7.34 (d, *J* = 8.8 Hz, 2H), 7.44 (s, 1H), 7.49 (t, *J* = 8.8 Hz, 1H), 7.63 (d, *J* = 8.8 Hz, 1H), 7.81 (d, *J* = 8.8 Hz, 2H), 10.11 (s, NH).

*2-Amino-3-methyl-N-(4-(2-oxopyridin-1(2H)-yl)phenyl)benzamide* (**5d**). Compound **5d** was prepared from **4d** according to the procedure described for the preparation of **5a**. White solid product (1.20 g, 90%). MS: [M + H]^+^ 320.04. ^1^H-NMR: δ ppm 2.12 (s, CH_3_), 6.13 (s, NH_2_), 6.30 (t, *J* = 6.8 Hz, 1H), 6.46 (d, *J* = 9.2 Hz, 1H), 6.58 (t, *J* = 7.2 Hz, 1H), 7.15 (d, *J* = 7.2 Hz, 1H), 7.34 (d, *J* = 8.8 Hz, 2H), 7.47–7.54 (m, 2H), 7.62 (d, *J* = 8.4 Hz, 1H), 7.82 (d, *J* = 8.8 Hz, 2H), 10.17 (s, NH).

*2-Amino-N-(4-(3-oxomorpholino)phenyl)benzamide* (**5e**). Compound **5e** was prepared from **4e** according to the procedure described for the preparation of **5a**. White solid product (1.21 g, 93%). MS: [M + H]^+^ 312.04. ^1^H-NMR: δ ppm 3.71 (t, *J* = 4.8 Hz, CH_2_), 3.96 (t, *J* = 4.8 Hz, CH_2_), 4.18 (s, CH_2_), 6.31 (s, NH_2_), 6.58 (t, *J* = 7.2 Hz, 1H), 6.74 (d, *J* = 8.0 Hz, 1H), 7.13 (t, *J* = 8.4 Hz, 1H), 7.33 (d, *J* = 7.2 Hz, 2H), 7.61 (d, *J* = 8.8 Hz, 1H), 7.72 (d, *J* = 8.8 Hz, 2H), 10.03 (s, NH).

*2-Amino-5-chloro-N-(4-(3-oxomorpholino)phenyl)benzamide* (**5f**). Compound **5f** was prepared from **4f** according to the procedure described for the preparation of **5a**. White solid product (1.28 g, 89%). MS: [M + H]^+^ 345.97. ^1^H-NMR: δ ppm 3.71 (t, *J* = 4.8 Hz, CH_2_), 3.96 (t, *J* = 4.8 Hz, CH_2_), 4.19 (s, CH_2_), 6.46 (s, NH_2_), 6.78 (d, *J* = 8.8 Hz, 1H), 7.23 (d, *J* = 8.8 Hz, 2H), 7.34 (d, *J* = 8.8 Hz, 2H), 7.68 (d, *J* = 7.6 Hz, 2H), 7.72 (s, 1H), 10.14 (s, NH).

*2-Amino-5-methyl-N-(4-(3-oxomorpholino)phenyl)benzamide* (**5g**). Compound **5g** was prepared from **4g** according to the procedure described for the preparation of **5a**. White solid product (1.22 g, 90%). MS: [M + H]^+^ 326.07. ^1^H-NMR: δ ppm 2.21 (s, CH_3_), 3.71 (t, *J* = 4.8 Hz, CH_2_), 3.96 (t, *J* = 4.8 Hz, CH_2_), 4.19 (s, CH_2_), 6.09 (s, NH_2_), 6.67 (d, *J* = 8.4 Hz, 1H), 7.03 (d, *J* = 8.0 Hz, 1H), 7.33 (d, *J* = 8.8 Hz, 2H), 7.42 (s, 1H), 7.72 (d, *J* = 8.8 Hz, 2H), 10.01 (s, NH).

*2-Amino-3-methyl-N-(4-(3-oxomorpholino)phenyl)benzamide* (**5h**). Compound **5h** was prepared from **4h** according to the procedure described for the preparation of **5a**. White solid product (1.23 g, 91%). MS: [M + H]^+^ 326.03. ^1^H-NMR: δ ppm 2.11 (s, CH_3_), 3.71 (t, *J* = 4.8 Hz, CH_2_), 3.96 (t, *J* = 4.8 Hz, CH_2_), 4.19 (s, CH_2_), 6.11 (s, NH_2_), 6.56 (t, *J* = 7.6 Hz, 1H), 7.13 (d, *J* = 6.8 Hz, 1H), 7.33 (d, *J* = 8.8 Hz, 2H), 7.51 (d, *J* = 7.6 Hz, 1H), 7.72 (d, *J* = 8.8 Hz, 2H), 10.06 (s, NH).

*5-Chloro-N-(2-((4-(2-oxopyridin-1(2H)-yl)phenyl)carbamoyl)phenyl)thiophene-2-carboxamide* (**1a**). To a stirred solution of **5a** (0.30 g, 0.98 mmol), K_2_CO_3_ (0.16 g, 1.18 mmol) and DMAP (0.01 g, 0.08 mmol) in THF (10 mL), solution of 5-chlorothiophene-2-carbonyl chloride (**6a**) (0.23 g, 1.27 mmol) in THF (5 mL) was added at room temperature and the mixture was refluxed for 4 h. The reaction mixture was cool down to room temperature and water (30 mL) was added. The resulting precipitate was collected by filtration. The authentic sample was prepared by recrystallization from DMF/MeOH. The reaction was monitored by TLC with EA. White solid product (0.27 g, 60%), m.p. > 250 °C. ^1^H-NMR: δ ppm 6.31 (t, *J* = 6.4 Hz, 1H), 6.47 (d, *J* = 9.2 Hz, 1H), 7.27 (d, *J* = 4.0 Hz, 1H), 7.32 (t, *J* = 7.2 Hz, 1H), 7.38 (d, *J* = 8.4 Hz, 2H), 7.50 (t, *J* = 8.8 Hz, 1H), 7.59–7.64 (m, 3H), 7.81 (d, *J* = 8.4 Hz, 2H), 7.92 (d, *J* = 7.6 Hz, 1H), 8.22 (d, *J* = 8.4 Hz, 1H), 10.67 (s, NH), 11.52 (s, NH). HRMS (ESI) calcd. for C_23_H_16_ClN_3_O_3_S: [M + Na]^+^ *m*/*z*: 472.0499, found: 472.0482.

*2-(4-Fluorobenzamido)-N-(4-(2-oxopyridin-1(2H)-yl)phenyl)benzamide* (**1b**). Compound **1b** was prepared from **5a** and 4-fluorobenzoyl chloride (**6b**) according to the procedure described for the preparation of **1a**. White solid product (0.24 g, 56%), m.p. > 250 °C. ^1^H-NMR: δ ppm 6.31 (t, *J* = 6.8 Hz, 1H), 6.47 (d, *J* = 8.8 Hz, 1H), 7.31 (t, *J* = 7.6 Hz, 1H), 7.37–7.42 (m, 4H), 7.50 (t, *J* = 8.8 Hz, 1H), 7.60–7.64 (m, 2H), 7.81 (d, *J* = 8.8 Hz, 2H), 7.93 (d, *J* = 7.6 Hz, 1H), 7.96–8.00 (m, 2H), 8.39 (d, *J* = 8.0 Hz, 1H), 10.68 (s, NH), 11.56 (s, NH). HRMS (ESI) calcd. for C_25_H_18_FN_3_O_3_: [M + Na]^+^ *m*/*z*: 450.1230, found: 450.1222.

*2-(4-Chlorobenzamido)-N-(4-(2-oxopyridin-1(2H)-yl)phenyl)benzamide* (**1c**). Compound **1c** was prepared from **5a** and 4-chlorobenzoyl chloride (**6c**) according to the procedure described for the preparation of **1a**. White solid product (0.28 g, 63%), m.p. > 250 °C. ^1^H-NMR: δ ppm 6.31 (t, *J* = 6.4 Hz, 1H), 6.47 (d, *J* = 9.2 Hz, 1H), 7.32 (t, *J* = 7.2 Hz, 1H), 7.38 (d, *J* = 8.8 Hz, 2H), 7.50 (t, *J* = 8.8 Hz, 1H), 7.60–7.65 (m, 4H), 7.81 (d, *J* = 8.8 Hz, 2H), 7.92 (d, *J* = 8.4 Hz, 3H), 8.38 (d, *J* = 8.0 Hz, 1H), 10.68 (s, NH), 11.57 (s, NH). HRMS (ESI) calcd. for C_25_H_18_ClN_3_O_3_: [M + Na]^+^ *m*/*z*: 466.0934, found: 466.0933.

*5-Chloro-N-(4-chloro-2-((4-(2-oxopyridin-1(2H)-yl)phenyl)carbamoyl)phenyl)thiophene-2-carboxamide* (**1d**). Compound **1d** was prepared from **5b** and **6a** according to the procedure described for the preparation of **1a**. White solid product (0.31 g, 65%), m.p. > 250 °C. ^1^H-NMR: δ ppm 6.31 (t, *J* = 6.8 Hz, 1H), 6.47 (d, *J* = 9.2 Hz, 1H), 7.27 (d, *J* = 4.0 Hz, 1H), 7.39 (d, *J* = 8.8 Hz, 2H), 7.50 (t, *J* = 8.8 Hz, 1H), 7.62–7.69 (m, 3H), 7.80 (d, *J* = 8.8 Hz, 2H), 7.96 (d, *J* = 2.4 Hz, 1H), 8.19 (d, *J* = 8.8 Hz, 1H), 10.73 (s, NH), 11.41 (s, NH). HRMS (ESI) calcd. for C_23_H_15_Cl_2_N_3_O_3_S: [M + Na]^+^ *m*/*z*: 506.0109, found: 506.0104.

*5-Chloro-2-(4-fluorobenzamido)-N-(4-(2-oxopyridin-1(2H)-yl)phenyl)benzamide* (**1e**). Compound **1e** was prepared from **5b** and **6b** according to the procedure described for the preparation of **1a**. White solid product (0.25 g, 55%), m.p. > 250 °C. ^1^H-NMR: δ ppm 6.30 (t, *J* = 6.8 Hz, 1H), 6.47 (d, *J* = 9.2 Hz, 1H), 7.40 (t, *J* = 8.8 Hz, 4H), 7.50 (t, *J* = 6.8 Hz, 1H), 7.62 (d, *J* = 1.6 Hz, 1H), 7.63 (d, *J* = 1.6 Hz, 1H), 7.68 (d, *J* = 8.8 Hz, 2H), 7.95–7.99 (m, 3H), 7.36 (d, *J* = 8.8 Hz, 1H), 10.74 (s, NH), 11.44 (s, NH). HRMS (ESI) calcd. for C_25_H_17_ClFN_3_O_3_: [M + Na]^+^ *m*/*z*: 484.0840, found: 484.0845.

*5-Chloro-2-(4-chlorobenzamido)-N-(4-(2-oxopyridin-1(2H)-yl)phenyl)benzamide* (**1f**). Compound **1f** was prepared from **5b** and **6c** according to the procedure described for the preparation of **1a**. White solid product (0.29 g, 60%), m.p. > 250 °C. ^1^H-NMR: δ ppm 6.30 (t, *J* = 6.0 Hz, 1H), 6.47 (d, *J* = 9.6 Hz, 1H), 7.39 (d, *J* = 8.4 Hz, 2H), 7.50 (t, *J* = 6.8 Hz, 1H), 7.62–7.70 (m, 4H), 7.78 (d, *J* = 8.0 Hz, 2H), 7.91 (d, *J* = 8.8 Hz, 2H), 7.97 (s, 1H), 8.34 (d, *J* = 9.6 Hz, 1H), 10.74 (s, NH), 11.45 (s, NH). HRMS (ESI) calcd. for C_25_H_17_Cl_2_N_3_O_3_: [M + Na]^+^ *m*/*z*: 500.0545, found: 500.0539.

*5-Chloro-N-(4-methyl-2-((4-(2-oxopyridin-1(2H)-yl)phenyl)carbamoyl)phenyl)thiophene-2-carboxami-de* (**1g**). Compound **1g** was prepared from **5c** and **6a** according to the procedure described for the preparation of **1a**. White solid product (0.27 g, 58%), m.p. > 250 °C. ^1^H-NMR: δ ppm 2.39 (s, CH_3_), 6.31 (t, *J* = 6.4 Hz, 1H), 6.47 (d, *J* = 9.2 Hz, 1H), 7.26 (d, *J* = 4 Hz, 1H), 7.25–7.43 (m, 3H), 7.50 (t, *J* = 8.8 Hz, 1H), 7.61–7.64 (m, 2H), 7.72 (s, 1H), 7.81 (d, *J* = 8.8 Hz, 2H), 8.08 (d, *J* = 8.4 Hz, 1H), 10.62 (s, NH), 11.38 (s, NH). HRMS (ESI) calcd. for C_24_H_18_ClN_3_O_3_S: [M + Na]^+^ *m*/*z*: 486.0655, found: 486.0648.

*2-(4-Fluorobenzamido)-5-methyl-N-(4-(2-oxopyridin-1(2H)-yl)phenyl)benzamide* (**1h**). Compound **1h** was prepared from **5c** and **6b** according to the procedure described for the preparation of **1a**. White solid product (0.27 g, 61%), m.p. > 250 °C. ^1^H-NMR: δ ppm 2.39 (s, CH_3_), 6.30 (t, *J* = 6.4 Hz, 1H), 6.47 (d, *J* = 9.2 Hz, 1H), 7.37–7.44 (m, 5H), 7.49 (t, *J* = 8.8 Hz, 1H), 7.62 (d, *J* = 5.6 Hz, 1H), 7.74 (s, 1H), 7.80 (d, *J* = 8.8 Hz, 2H), 7.94–7.98 (m, 2H), 8.27 (d, *J* = 8.4 Hz, 1H), 10.64 (s, NH), 11.43 (s, NH). HRMS (ESI) calcd. for C_26_H_20_FN_3_O_3_: [M + Na]^+^ *m*/*z*: 464.1386, found: 464.1379.

*2-(4-Chlorobenzamido)-5-methyl-N-(4-(2-oxopyridin-1(2H)-yl)phenyl)benzamide* (**1i**). Compound **1i** was prepared from **5c** and **6c** according to the procedure described for the preparation of **1a**. White solid product (0.29 g, 63 %), m.p. > 250 °C. ^1^H-NMR: δ ppm 2.39 (s, CH_3_), 6.30 (t, *J* = 7.2 Hz, 1H), 6.47 (d, *J* = 9.2 Hz, 1H), 7.38 (d, *J* = 8.8 Hz, 2H), 7.43 (d, *J* = 8.4 Hz, 1H), 7.50 (t, *J* = 8.8 Hz, 1H), 7.62–7.64 (m, 3H), 7.73 (s, 1H), 7.80 (d, *J* = 8.8 Hz, 2H), 7.91 (d, *J* = 8.8 Hz, 2H), 8.25 (d, *J* = 8.4 Hz, 1H), 10.64 (s, NH), 11.44 (s, NH). HRMS (ESI) calcd. for C_26_H_20_ClN_3_O_3_: [M + Na]^+^ *m*/*z*: 480.1091, found: 480.1083.

*5-Chloro-N-(2-methyl-6-((4-(2-oxopyridin-1(2H)-yl)phenyl)carbamoyl)phenyl)thiophene-2-carboxami-de* (**1j**). Compound **1j** was prepared from **5d** and **6a** according to the procedure described for the preparation of **1a**. White solid product (0.23 g, 50%), m.p. > 250 °C. ^1^H-NMR: δ ppm 2.27 (s, CH_3_), 6.28 (t, *J* = 6.8 Hz, 1H), 6.45 (d, *J* = 9.2 Hz, 1H), 7.22 (d, *J* = 4.0 Hz, 1H), 7.30–7.34 (m, 2H), 7.37 (d, *J* = 7.6 Hz, 1H), 7.46–7.52 (m, 3H), 7.59 (d, *J* = 6.8 Hz, 1H), 7.77 (d, *J* = 8.8 Hz, 2H), 7.84 (d, *J* = 4.4 Hz, 1H), 10.10 (s, NH), 11.42 (s, NH). HRMS (ESI) calcd. for C_24_H_18_ClN_3_O_3_S: [M + Na]^+^ *m*/*z*: 486.0655, found: 486.0649.

*2-(4-Fluorobenzamido)-3-methyl-N-(4-(2-oxopyridin-1(2H)-yl)phenyl)benzamide* (**1k**). Compound **1k** was prepared from **5d** and **6b** according to the procedure described for the preparation of **1a**. White solid product (0.22 g, 46%), m.p. > 250 °C. ^1^H-NMR: δ ppm 2.28 (s, CH_3_), 6.27 (t, *J* = 6.8 Hz, 1H), 6.44 (d, *J* = 9.2 Hz, 1H), 7.29–7.32 (m, 5H), 7.46–7.50 (m, 3H), 7.57 (d, *J* = 8.8 Hz, 1H), 7.77 (d, *J* = 8.8 Hz, 2H), 7.98–8.01 (m, 2H), 9.99 (s, NH), 11.41 (s, NH). HRMS (ESI) calcd. for C_26_H_20_FN_3_O_3_: [M + Na]^+^ *m*/*z*: 464.1386, found: 464.1381.

*2-(4-Chlorobenzamido)-3-methyl-N-(4-(2-oxopyridin-1(2H)-yl)phenyl)benzamide* (**1l**). Compound **1l** was prepared from **5d** and **6c** according to the procedure described for the preparation of **1a**. White solid product (0.22 g, 48%), m.p. > 250 °C. ^1^H-NMR: δ ppm 2.28 (s, CH_3_), 6.27 (t, *J* = 6.4 Hz, 1H), 6.44 (d, *J* = 8.8 Hz, 1H), 7.30 (d, *J* = 8.8 Hz, 2H), 7.36 (t, *J* = 7.6 Hz, 1H), 7.46–7.50 (m, 3H), 7.56–7.59 (m, 3H), 7.77 (d, *J* = 8.4 Hz, 2H), 7.94 (d, *J* = 8.4 Hz, 2H), 10.05 (s, NH), 10.42 (s, NH). HRMS (ESI) calcd. for C_26_H_20_ClN_3_O_3_: [M + Na]^+^ *m*/*z*: 480.1091, found: 480.1087.

*5-Chloro-N-(2-((4-(3-oxomorpholino)phenyl)carbamoyl)phenyl)thiophene-2-carboxamide* (**1m**). Compound **1m** was prepared from **5e** and **6a** according to the procedure described for the preparation of **1a**. White solid product (0.29 g, 63%), m.p. > 250 °C. ^1^H-NMR: δ ppm 3.72 (t, *J* = 4.8 Hz, CH_2_), 3.97 (t, *J* = 4.8 Hz, CH_2_), 4.19 (s, CH_2_), 7.27 (d, *J* = 4.0 Hz, 1H), 7.30 (t, *J* = 7.2 Hz, 1H), 7.38 (d, *J* = 8.8 Hz, 2H), 7.58–7.60 (m, 2H), 7.72 (d, *J* = 8.8 Hz, 2H), 7.91 (d, *J* = 7.6 Hz, 1H), 8.24 (d, *J* = 8.4 Hz, 1H), 10.57 (s, NH), 11.60 (s, NH). HRMS (ESI) calcd. for C_22_H_18_ClN_3_O_4_S: [M + Na]^+^ *m*/*z*: 478.0604, found: 478.0613.

*2-(4-Fluorobenzamido)-N-(4-(3-oxomorpholino)phenyl)benzamide* (**1n**). Compound **1n** was prepared from **5e** and **6b** according to the procedure described for the preparation of **1a**. White solid product (0.27 g, 62%), m.p. > 250 °C. ^1^H-NMR: δ ppm 3.72 (t, *J* = 4.8 Hz, CH_2_), 3.97 (t, *J* = 4.8 Hz, CH_2_), 4.19 (s, CH_2_), 7.29 (t, *J* = 7.6 Hz, 1H), 7.36–7.42 (m, 4H), 7.61 (t, *J* = 7.8 Hz, 1H), 7.71 (d, J = 8.8 Hz, 2H), 7.92 (d, *J* = 8.0 Hz, 1H), 7.95–7.99 (m, 2H), 8.42 (d, *J* = 8.4 Hz, 1H), 10.58 (s, NH), 11.64 (s, NH). HRMS (ESI) calcd. for C_24_H_20_FN_3_O_4_: [M + Na]^+^ *m*/*z*: 456.1336, found: 456.1386.

*2-(4-Chlorobenzamido)-N-(4-(3-oxomorpholino)phenyl)benzamide* (**1o**). Compound **1o** was prepared from **5e** and **6c** according to the procedure described for the preparation of **1a**. White solid product (0.30 g, 67%), m.p. > 250 °C. ^1^H-NMR: δ ppm 3.59 (t, *J* = 4.8 Hz, CH_2_), 3.71 (t, *J* = 4.8 Hz, CH_2_), 4.19 (s, CH_2_), 7.30 (t, *J* = 7.2 Hz, 1H), 7.37 (d, *J* = 8.8 Hz, 2H), 7.60–7.65 (m, 3H), 7.71 (d, *J* = 9.2 Hz, 2H), 7.91 (d, *J* = 8.4 Hz, 3H), 8.40 (d, *J* = 8.0 Hz, 1H), 10.58 (s, NH), 11.64 (s, NH). HRMS (ESI) calcd. for C_24_H_20_ClN_3_O_4_: [M + Na]^+^ *m*/*z*: 472.1040, found: 472.1035.

*5-Chloro-N-(4-chloro-2-((4-(3-oxomorpholino)phenyl)carbamoyl)phenyl)thiophene-2-carboxamide* (**1p**). Compound **1p** was prepared from **5f** and **6a** according to the procedure described for the preparation of **1a**. White solid product (0.32 g, 65%), m.p. > 250 °C. ^1^H-NMR: δ ppm 3.72 (t, *J* = 4.8 Hz, CH_2_), 3.97 (t, *J* = 4.8 Hz, CH_2_), 4.19 (s, CH_2_), 7.26 (s, 1H), 7.38 (d, *J* = 8.8 Hz, 2H), 7.62–7.71 (m, 4H), 7.95 (d, *J* = 2.0 Hz, 1H), 8.22 (d, *J* = 8.8 Hz, 1H), 10.63 (s, NH), 11.49 (s, NH). HRMS (ESI) calcd. for C_22_H_17_Cl_2_N_3_O_4_S: [M + Na]^+^ *m*/*z*: 512.0215, found: 512.0205.

*5-Chloro-2-(4-fluorobenzamido)-N-(4-(3-oxomorpholino)phenyl)benzamide* (**1q**). Compound **1q** was prepared from **5f** and **6b** according to the procedure described for the preparation of **1a**. White solid product (0.29 g, 63%), m.p. > 250 °C. ^1^H-NMR: δ ppm 3.71 (t, *J* = 4.8 Hz, CH_2_), 3.97 (t, *J* = 4.8 Hz, CH_2_), 4.19 (s, CH_2_), 7.33–7.43 (m, 4H), 7.65–7.70 (m, 3H), 7.95–7.98 (m, 3H), 8.39 (d, *J* = 8.8 Hz, 1H), 10.65 (s, NH), 11.54 (s, NH). HRMS (ESI) calcd. for C_24_H_19_ClFN_3_O_4_: [M + Na]^+^ *m*/*z*: 490.0946, found: 490.0940.

*5-Chloro-2-(4-chlorobenzamido)-N-(4-(3-oxomorpholino)phenyl)benzamide* (**1r**). Compound **1r** was prepared from **5f** and **6c** according to the procedure described for the preparation of **1a**. White solid product (0.32 g, 66%), m.p. > 250 °C. ^1^H-NMR: δ ppm 3.71 (t, *J* = 4.8 Hz, CH_2_), 3.97 (t, *J* = 4.8 Hz, CH_2_), 4.19 (s, CH_2_), 7.37 (d, *J* = 8.8 Hz, 2H), 7.63 (d, *J* = 8.4 Hz, 2H), 7.67–7.72 (m, 3H), 7.91 (d, *J* = 8.4 Hz, 2H), 7.96 (d, *J* = 2.4 Hz, 1H), 8.37 (d, *J* = 8.8 Hz, 1H), 10.65 (s, NH), 11.52 (s, NH). HRMS (ESI) calcd. for C_24_H_19_Cl_2_N_3_O_4_: [M + Na]^+^ *m*/*z*: 506.0650, found: 506.0644.

*5-Chloro-N-(4-methyl-2-((4-(3-oxomorpholino)phenyl)carbamoyl)phenyl)thiophene-2-carboxamide* (**1s**). Compound **1s** was prepared from **5g** and **6a** according to the procedure described for the preparation of **1a**. White solid product (0.30 g, 63%), m.p. > 250 °C. ^1^H-NMR: δ ppm 2.37 (s, CH_3_), 3.72 (t, *J* = 4.8 Hz, CH_2_), 3.97 (t, *J* = 4.8 Hz, CH_2_), 4.19 (s, CH_2_), 7.25 (d, *J* = 3.6 Hz, 1H), 7.36–7.42 (m, 3H), 7.60 (d, *J* = 3.2 Hz, 1H), 7.70 (d, *J* = 8.8 Hz, 3H), 8.10 (d, *J* = 8.0 Hz, 1H), 10.52 (s, NH), 11.45 (s, NH). HRMS (ESI) calcd. for C_23_H_20_ClN_3_O_4_S: [M + Na]^+^ *m*/*z*: 492.0761, found: 492.0754.

*2-(4-Fluorobenzamido)-5-methyl-N-(4-(3-oxomorpholino)phenyl)benzamide* (**1t**). Compound **1t** was prepared from **5g** and **6b** according to the procedure described for the preparation of **1a**. White solid product (0.28 g, 63%), m.p. > 250 °C. ^1^H-NMR: δ ppm 2.38 (s, CH_3_), 3.72 (t, *J* = 4.8 Hz, CH_2_), 3.97 (t, *J* = 4.8 Hz, CH_2_), 4.19 (s, CH_2_), 7.36–7.43 (m, 5H), 7.69–7.73 (m, 3H), 7.94–7.97 (m, 2H), 8.29 (d, *J* = 8.4 Hz, 1H), 10.54 (s, NH), 11.49 (s, NH). HRMS (ESI) calcd. for C_25_H_22_FN_3_O_4_: [M + Na]^+^ *m*/*z*: 470.1492, found: 470.1487.

*2-(4-Chlorobenzamido)-5-methyl-N-(4-(3-oxomorpholino)phenyl)benzamide* (**1u**). Compound **1u** was prepared from **5g** and **6c** according to the procedure described for the preparation of **1a**. White solid product (0.28 g, 60%), m.p. > 250 °C. ^1^H-NMR: δ ppm 2.39 (s, CH_3_), 3.72 (t, *J* = 4.8 Hz, CH_2_), 3.97 (t, *J* = 4.8 Hz, CH_2_), 4.19 (s, CH_2_), 7.37 (d, *J* = 8.8 Hz, 2H), 7.42 (d, *J* = 8.0 Hz, 1H), 7.63 (d, *J* = 8.4 Hz, 2H), 7.69–7.73 (m, 2H), 7.90 (d, *J* = 8.8 Hz, 2H), 8.27 (d, *J* = 8.4 Hz, 1H), 10.54 (s, NH), 11.51 (s, NH). HRMS (ESI) calcd. for C_25_H_22_ClN_3_O_4_: [M + Na]^+^ *m*/*z*: 486.1197, found: 486.1185.

*5-Chloro-N-(2-methyl-6-((4-(3-oxomorpholino)phenyl)carbamoyl)phenyl)thiophene-2-carboxamide* (**1v**). Compound **1v** was prepared from **5h** and **6a** according to the procedure described for the preparation of **1a**. White solid product (0.24 g, 51%), m.p. > 250 °C. ^1^H-NMR: δ ppm 2.22 (s, CH_3_), 3.68 (t, *J* = 4.8 Hz, CH_2_), 3.95 (t, *J* = 4.8 Hz, CH_2_), 4.17 (s, CH_2_), 7.24 (d, *J* = 7.6 Hz, 1H), 7.31 (d, *J* = 9.2 Hz, 2H), 7.39 (t, *J* = 8.8 Hz, 1H), 7.55 (d, *J* = 8.8 Hz, 1H), 7.6 (d, *J* = 8.8 Hz, 2H), 7.70–7.79 (m, 2H), 10.10 (s, NH), 10.29 (s, NH). HRMS (ESI) calcd. for C_23_H_20_ClN_3_O_4_S: [M + Na]^+^ *m*/*z*: 492.0761, found: 492.0755.

*2-(4-Fluorobenzamido)-3-methyl-N-(4-(3-oxomorpholino)phenyl)benzamide* (**1w**). Compound **1w** was prepared from **5h** and **6b** according to the procedure described for the preparation of **1a**. White solid product (0.19 g, 43%), m.p. > 250 °C. ^1^H-NMR: δ ppm 2.49 (s, CH_3_), 3.67 (t, *J* = 4.8 Hz, CH_2_), 3.94 (t, *J* = 4.8 Hz, CH_2_), 4.16 (s, CH_2_), 7.27–7.36 (m, 5H), 7.47 (t, *J* = 7.6 Hz, 2H), 7.67 (d, *J* = 8.4 Hz, 2H), 7.97–8.01 (m, 2H), 9.98 (s, NH), 10.28 (s, NH). HRMS (ESI) calcd. for C_25_H_22_FN_3_O_4_: [M + Na]^+^ *m*/*z*: 470.1492, found: 470.1488.

*2-(4-Chlorobenzamido)-3-methyl-N-(4-(3-oxomorpholino)phenyl)benzamide* (**1x**). Compound **1x** was prepared from **5h** and **6c** according to the procedure described for the preparation of **1a**. White solid product (0.21 g, 46%), m.p. > 250 °C. ^1^H-NMR: δ ppm 2.27 (s, CH_3_), 3.67 (t, *J* = 4.8 Hz, CH_2_), 3.95 (t, *J* = 4.8 Hz, CH_2_), 4.17 (s, CH_2_), 7.28 (d, *J* = 8.8 Hz, 2H), 7.34 (t, *J* = 6.8 Hz, 1H), 7.47 (t, *J* = 8.4 Hz, 2H), 7.56 (d, *J* = 8.4 Hz, 2H), 7.67 (d, *J* = 8.4 Hz, 2H), 7.93 (d, *J* = 8.0 Hz, 2H), 10.03 (s, NH), 10.29 (s, NH). HRMS (ESI) calcd. for C_25_H_22_ClN_3_O_4_: [M + Na]^+^ *m*/*z*: 486.1197, found: 486.1196.

### 3.3. Inhibition of FXa in Vitro

The inhibition of FXa was measured using human FXa (Hyphen BioMed, Paris, France) and chromogenic substrate CS-11(22) (Hyphen BioMed, Paris, France) in 384-well microtiter plates at room temperature. The synthesized compounds (**1a**–**1x**) and Rivaroxaban were dissolved in DMSO at a concentration of 10 mM and then serially diluted to spanning a range of 30 nM to 100 μM, respectively. 2 μL of FXa (56.8 nM), 16 μL of Tris buffer (adjust to pH 7.4 with HCl containing 0.3 M NaCl and 50 mM Tris) and 3 μL of test compound were added to the well, respectively. The negative control was composed of the same mixed solutions except replacing test compound with DMSO. The positive control was composed of the same mixed solutions except replacing test compound with rivaroxaban. After incubated at 37 °C for 5 min, 8 μL of FXa substrate solution (3.5 mM) was added and then incubated at 37 °C for 25 min. The FXa activity was measured at 405 nm using a SpectraMax M5 (Molecular Devices, Sunnyvale, CA, USA). The IC_50_ was calculated by the software named SPSS (IBM, North Castle, NY, USA) and the Probit function in it.

### 3.4. Thrombin Inhibition in Vitro of ***1a***, ***1g*** and ***1s***

The inhibition of thrombin was measured using human FIIa (Hyphen BioMed, Paris, France) and chromogenic substrate CS-01(38) (Hyphen BioMed, Paris, France) in 384-well microtiter plates at room temperature. The compounds **1a**, **1g** and **1s** and Rivaroxaban were dissolved in DMSO to a concentration of 10 mM and then serially diluted to spanning a range of 10 μM to 100 μM, respectively. 2 μL of FIIa (3 NIH/mL), 20 μL of Tris buffer (adjust to pH 7.4 with HCl) containing 0.3 M NaCl and 50 mM Tris and 2 μL of test compound were added to the well, respectively. The negative control was composed of the same mixed solutions except replacing test compound with DMSO. The positive control was composed of the same mixed solutions except replacing test compound with Rivaroxaban. After incubated at 37 °C for 5 min, 3 μL of FIIa substrate solution (4 mM) was added and then incubated at 37 °C for 25 min. The FIIa activity was measured at 405 nm using a SpectraMax M5 (Molecular Devices).

### 3.5. Prothrombin Time (PT) Assay

A commercially available automatic coagulometer (Steellex Science Instrument Co., Ltd., Beijing, China) was employed to measure PT. The clotting times were also measured using the instrument itself, in accordance with the manufacturer’s instructions. Increasing concentrations of inhibitor or solvent were added to rat (Sprague-Dawley rats, Shanchuanhong Experimental Animals Co., Ltd., Tianjin, China) and human (29 Years old, male, Chinese) plasma and incubated for 3 min at 37 °C. Prothrombin time (PT) was determined by automatic coagulometer.

### 3.6. Docking Simulation Study

FXa structure was selected from the protein data bank (PDB code: 2xbv) and prepared using Protein Preparation Wizard in Schrödinger package, including assigning bond orders, adding hydrogen atoms, deleting water molecules, creating disulfide bonds and capping terminals. The original ligand of the protein structure-XBV was used as the docking center to generate the receptor grid parameters. The box size was set as 12 Å. Compounds **1g** and **1s** were prepared using the LigPrep module in Schrödinger. Epik method was used to determine possible ionization state of ligands at pH 7.0 ± 2.0 and low-energy conformers were produced using OPLS-2005 force field. Molecular docking calculations were performed by using Glide module with default parameters at standard precision in Schrödinger.

## 4. Conclusions 

In this study, we designed and synthesized a series of novel potent FXa inhibitors based on the anthranilamide scaffold. *In vitro* inhibition activity studies showed that compounds **1a**, **1g** and **1s** displayed evident FXa inhibition and excellent selectivity over thrombin. Compounds **1g** and **1s** also exhibited pronounced anticoagulant activity in *in vitro* anticoagulant activity studies. Further docking simulation study also disclosed that the interaction of compounds **1g** and **1s** with FXa was very similar to that of rivaroxaban. Therefore, compounds **1g** and **1s** with an anthranilamide scaffold could be considered as lead compounds for exploring new FXa inhibitors with better medicinal effects and further modification of this structure for better antithrombotic activity is still in progress.

## Figures and Tables

**Table 1:** The structures of the target compounds **1a**–**1x** and their biological activity evaluation. 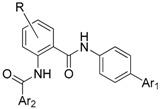

Compound	R	Ar_1_	Ar_2_	IC_50_ (nM)
**1a**	H	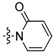	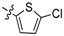	30.2
**1b**	H	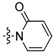	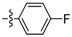	>1000
**1c**	H	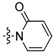	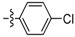	197.3
**1d**	5-chloro	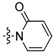	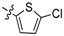	430.1
**1e**	5-chloro	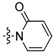	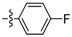	>1000
**1f**	5-chloro	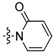	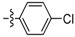	570.5
**1g**	5-methyl	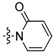	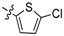	25.0
**1h**	5-methyl	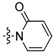	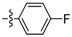	>1000
**1i**	5-methyl	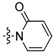	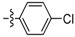	510.8
**1j**	3-methyl	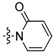	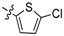	>1000
**1k**	3-methyl	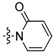	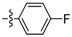	>1000
**1l**	3-methyl	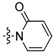	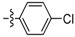	>1000
**1m**	H	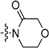	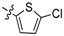	317.4
**1n**	H	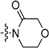	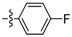	>1000
**1o**	H	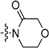	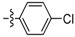	439.6
**1p**	5-chloro	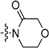	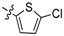	110.7
**1q**	5-chloro	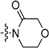	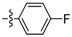	>1000
**1r**	5-chloro	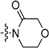	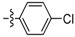	951.3
**1s**	5-methyl	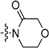	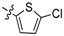	71.0
**1t**	5-methyl	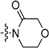	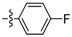	>1000
**1u**	5-methyl	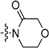	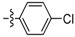	>1000
**1v**	3-methyl	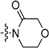	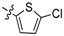	>1000
**1w**	3-methyl	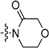	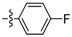	>1000
**1x**	3-methyl	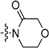	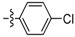	>1000
Rivaroxaban				0.9

**Table 2:** The anticoagulant activity of **1g** and **1s**.

Compound	2 × PT (μM) (Rat)	2 × PT (μM) (Human)
**1g**	19.7	12.8
**1s**	24.2	10.4
Rivaroxaban	0.3	0.2
